# Impact of chronic alcohol exposure on conventional and regulatory murine T cell subsets

**DOI:** 10.3389/fimmu.2023.1142614

**Published:** 2023-03-17

**Authors:** Cameron W. Paterson, Melissa B. Gutierrez, Craig M. Coopersmith, Mandy L. Ford

**Affiliations:** ^1^ Department of Surgery, Emory University School of Medicine, Atlanta, GA, United States; ^2^ Medical Corps, United States Navy, Navy Reserve Officer Training Corps (NROTC), Atlanta, GA, United States; ^3^ Emory Critical Care Center, Emory University School of Medicine, Atlanta, GA, United States; ^4^ Emory Transplant Center, Emory University School of Medicine, Atlanta, GA, United States

**Keywords:** T cell, regulatory T cell, alcohol, T cell activation, effector T cell

## Abstract

**Introduction:**

Chronic alcohol use poses significant negative consequences to public health and, among its many biologic effects, is associated with significant T cell dysregulation within the adaptive immune system that has yet to be fully characterized. Novel, automated strategies for high dimensional flow cytometric analysis of the immune system are rapidly improving researchers’ ability to detect and characterize rare cell types.

**Methods:**

Using a murine model of chronic alcohol ingestion in conjunction with viSNE and CITRUS analysis tools, we performed a machine-driven, exploratory analysis comparing rare splenic subpopulations within the conventional CD4^+^, regulatory CD4^+^ and CD8^+^ T cell compartments between alcohol- and water-fed animals.

**Results:**

While there were no differences in the absolute numbers of bulk CD3^+^ T cells, bulk CD4^+^ T cells, bulk CD8^+^ T cells, Foxp3^-^ CD4^+^ conventional T cells (T_conv_) or Foxp3^+^ CD4^+^ regulatory T cells (T_reg_), we identified populations of naïve Helios^+^ CD4^+^T_conv_ and naïve CD103^+^ CD8^+^ splenic T cells that were decreased in chronically alcohol exposed mice versus water-fed controls. In addition, we identified increased CD69^+^ Treg and decreased CD103^+^ effector regulatory T cell (eT_reg_) subsets in conjunction with increased frequency of a population that may represent a transitional phenotype between central regulatory T cell (cT_reg_) and eT_reg_.

**Discussion:**

These data provide further resolution into the character of decreased naïve T cell populations known to be present in alcohol exposed mice, as well as describe alterations in effector regulatory T cell phenotypes associated with the pathogenesis of chronic alcohol-induced immune dysfunction.

## Introduction

Alcohol use disorder (AUD) affects approximately 33 million individuals in the United States (1) and excessive alcohol consumption is a leading cause of premature mortality accounting for 1-in-10 deaths among working-age adults (2). Among its numerous effects, chronic alcohol exposure is associated with a variety of inflammatory, infectious and malignant pathologies that reflect both pro- and anti-inflammatory immune dysregulation (3). Our laboratory has investigated adaptive immune responses in murine models of chronic alcohol exposure followed by sepsis, and reported that alcohol-drinking mice exhibit increased sepsis mortality as compared to water-drinking mice (4). Specifically, we reported a number of T cell-specific perturbations, including altered subset frequencies (4), impaired activation (5), and enhanced proinflammatory cytokine release (5, 6). Other investigators have also noted T cell alterations in response to chronic alcohol exposure alone (7), including significant leukopenia in both humans (8) and rodents (9, 10) resulting in T cell homeostatic proliferation and, secondarily, an increase in peripheral memory T cells and a decrease in naïve T cells (11–14). Similarly, T cells of alcohol drinking humans (12, 15, 16) and mice (13, 14) show increased activation and inflammatory cytokine release consistent with a host phenotype marked by chronic inflammation (7). T cell subsets show differential responses to chronic alcohol exposure, with studies demonstrating loss of CD4^+^ T cells (17, 18) and overactivation of CD8^+^ T cells (18), as well as decreased numbers of regulatory T cells (T_reg_) in the dermis (19). Investigators have also reported that alcohol exposure impairs murine T cell tissue extravasation in response to inflammatory stimuli (10, 20), while *in vivo* (21) and *in vitro* (21, 22) human studies have shown alcohol-exposed T cells to undergo enhanced activation-induced cell death (23). Alcohol has additionally been shown to interfere with thymocyte development (7) and to predispose CD4^+^ T cells to Th2 polarization and suppress Th1 and Th17 responses (24). T cell-to-antigen presenting cell (APC) signaling is also impaired as a result of co-stimulatory molecule downregulation following alcohol exposure (13, 24, 25).

Flow cytometry is an indispensable tool for characterizing immune cells (26) that traditionally relies on biaxial plots to visualize expression of up to two parameters simultaneously, on which gates can be drawn to delineate specific subpopulations (26). However, this approach has critical limitations including logistical barriers to manual analysis of all possible comparison permutations, particularly as new generations of cytometers facilitate measurement of over twenty parameters simultaneously (26). Additionally, user-driven gating approaches rely on prior knowledge of anticipated subpopulations is itself inherently biased (26). Recent advances in cytometry, however, have introduced a series of computational analysis approaches that bypass manual gating and allow for unbiased, high dimensional analysis of single-cell cytometry data (26). These approaches can be categorized by their degree of supervision (supervised *vs*. unsupervised) and use of a clustering versus dimensionality-reduction strategy, which can be further classified as linear or nonlinear (27). T-stochastic neighbor embedding (t-SNE) (28) and its derivative used for data visualization-viSNE (29)-is a popular nonlinear algorithm that analyzes the similarity of cells in high-dimension before reducing them into an easily visualized two-dimensional scatter plot where their spatial proximity is reflective of their high dimensional relationship and allows for visualization of subpopulations as small as 0.25% (26). Cluster identification, characterization, and regression (CITRUS) (30) is an unsupervised clustering-based algorithm (26, 27, 31) that hierarchically groups phenotypically similar cells together into clusters with a minimum frequency threshold set by the user followed by calculation of cluster characteristics and, finally, a regularized classification model to identify stratifying clusters that predict a user-defined experimental endpoint (26).

Our laboratory has previously used a predecessor to CITRUS - spanning-tree progression analysis of density-normalized events (SPADE)- that follows similar principles but lacks the ability to compare experimental endpoints (27) in order perform high dimensional characterization of CD4^+^ T cells in tumor-bearing mice subject to sepsis (32). Similarly, other groups have effectively utilized CITRUS in a variety of disease-specific applications for T cell analysis (33–36). Here, we sought to apply high dimensional analysis techniques to characterize T cell alterations resulting from chronic alcohol exposure and explore the effect of chronic alcohol exposure on both effector and regulatory T cell subsets using this machine-based analysis strategy. We aimed to characterize rare T cell subpopulations in the CD8^+^, conventional CD4^+^ (CD4^+^ T_conv_) and regulatory CD4^+^ (CD4^+^ Treg) compartments in a murine model of chronic alcohol exposure using an iterative series of viSNE and CITRUS analyses and multimodal data visualization strategies similar to those described by Polikowsky et al. (33). To accomplish this, we employed an exploratory panel of T cell markers, including those delineating lineage (CD3, CD4, CD8, Foxp3), trafficking behavior (CCR4, CD103, CD62L), activation (CD25, CD69, Helios), co-stimulation (CD28, GITR, ICOS), co-inhibition (CTLA-4, KLRG1) immunologic memory (CD44, Ly6C), and proliferation (Ki67).

## Materials and methods

### Animals

Male and female 6-week-old B6 mice were purchased from Charles River Laboratories. This study was approved by the Emory University Institutional Animal Care and Use Committee (IACUC) [Protocol: PROTO201800161] and animal care was performed in accordance with all relevant IACUC and federal rules and guidelines. Animals randomized to either water (H2O) or alcohol (EtOH) drinking groups. Animals were sacrificed by isofluorane inhalation plus cervical dislocation at the conclusion of the 12-week drinking period for splenocyte analysis.

### Chronic alcohol ingestion model

Animals were equally randomized to receive either a water or alcohol diet. Animals in the alcohol arm received increasing concentrations of alcohol-in-water from 0% to 20% (by volume) over a two-week period (5% for 5 days, 10% for 5 days, 15% for 5 days), followed by 20% alcohol in water for ten additional weeks with weekly replacement of the alcohol solution. Water drinking animals received standard drinking water for an equivalent duration of time. Previous work from our lab has demonstrated that this protocol does not alter liver histology, renal function (6, 37), or body weight (4), and achieves a blood alcohol concentration (BAC) of 28mg/dl (4), which is approximately the BAC achieved in a 150 lb. person after one alcoholic drink.

### Flow cytometry

Animals were sacrificed and their spleens harvested at the conclusion of the 12-week drinking protocol. Splenocytes were first treated with Fc blocking agent (TruStain FcX, Biolegend). Surface staining was performed using anti-CD4-BUV395 (GK1.5, BD), anti-CD3-BUV496 (145-2c11, BD), anti-CD8-BUV737 (53-67, BD), anti-CD44-BUV805 (IM7, BD), anti-CCR4-eFluor450 (2G12, Biolegend), anti-Ly6C-BV510 (HK1.4, Biolegend), anti-CD103-BV605 (2E7, Biolegend), anti-CD69-BV650 (H1.2F3, Biolegend), anti-ICOS-BV711 (C398.4A, Biolegend), anti-GITR-BV786 (DTA-1, BD), anti-KLRG1-FITC (2F1/KLRG1, Biolegend), anti-CD62L-PE-Dazzle (MEL-14, Biolegend), anti-CD28-PE-Cy7 (E18, Biolegend), and anti-CD25-APC-Cy7 (PC61, Biolegend). Cells were then fixed and permeabilized (Foxp3/Transcription Factor Fixation/Permeabilization Concentrate and Diluent, eBioscience).

Intracellular/intranuclear staining was performed using the BD Foxp3 Kit per manufacturer’s instruction and anti-Helios-PerCP-Cy5.5 (22F6, Biolegend), anti-CTLA-4-PE (UC10-4B9, Biolegend), anti-Foxp3-APC (FJK-16s, eBioscience), and anti-Ki67-Alexa700 (16A8, Biolegend). CTLA-4 was measured as an intracellular stain because it is rapidly internalized/recycled on the plasma membrane, making detection of the surface protein difficult (38–40). Accucheck Counting Beads (Thermo Fisher Scientific) were added to calculate absolute T cell numbers per spleen. An LSRFortessa flow cytometer (BD Biosciences) was used to collect all samples. Data was analyzed using FlowJo v10.6 software (FlowJo, LLC) prior to transfer to the Cytobank platform (Cytobank.org).

### Cytobank analysis

Traditional flow cytometry gating techniques were applied to isolate lymphocytes, followed by single cells and CD3^+^ T cells using FlowJo v10.6 (FlowJo, LLC). CD3^+^ T cells were then exported as new FCS files with applied internal compensation and uploaded to the Cytobank (Cytobank.org) platform (31). Within Cytobank, the following protocol was followed using strategies adapted from Polikowsky et al. (33).

#### Data preparation

**Figure 1:**
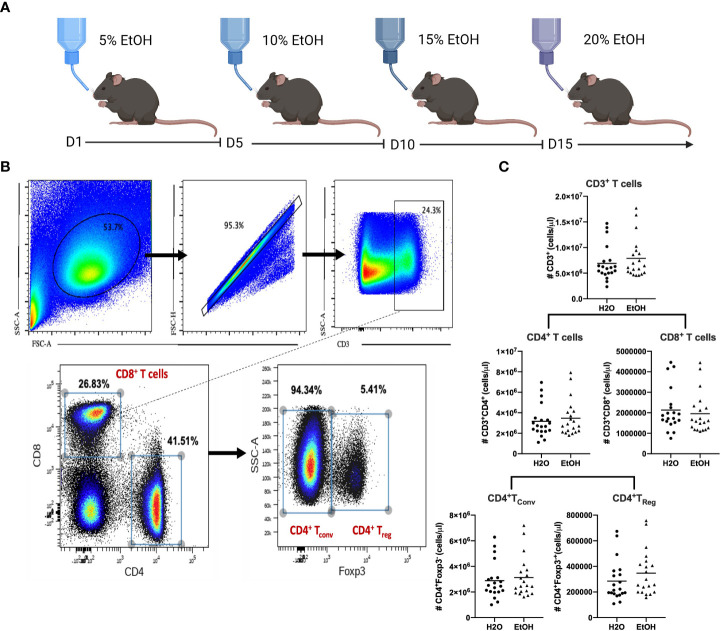
Chronic alcohol exposure does not impact absolute numbers of CD4^+^ or CD8^+^ T cells. **(A)**, Mice (n=20/group) were subjected to 12-weeks of an alcohol or water diet as described in Materials and Methods followed by sacrifice and collection of splenocytes for flow cytometry. **(B)**, Gating strategy to identify conventional CD4^+^ and CD8^+^ T cells as well as Foxp3^+^ Treg. **(C)**, The number of CD3^+^ T cells, CD8^+^ T cells, CD4^+^ T cells, CD4^+^T_conv_ or CD4^+^T_Reg_ did not differ between water- *vs*. alcohol-exposed mice. Data were tested for normality and compared with either *t* test or Mann-Whitney test.

Samples were examined for data tidying and quality control. Arcsinh transformations were applied and scaling adjusted to achieve appropriate marker display on each channel. Staining was examined to confirm presence of a positive signal for each channel. *Pre-gating.* Within Cytobank, CD3^+^ cells were further manually gated to facilitate downstream analysis of three distinct T cell populations: 1. CD8^+^CD4^-^ (CD8^+^ T cells), 2. CD8^-^CD4^+^Foxp3^-^ (CD4^+^ T_Conv_), and 3. CD8^-^CD4^+^Foxp3^+^ (CD4^+^ T_reg_) (as shown in **Figure 1**).

#### Data visualization using viSNE

viSNE analysis was performed for each of the three T cell populations. For analysis of CD8^+^ T cells and CD4^+^ T_Conv_, downsampling was performed to 20,000 events per sample to allow detection of subpopulations as small as 2% with a 5% coefficient of variation (CV) (33, 41). CD4^+^ T_reg_ were downsampled to 4000 events to allow detection of 10% subpopulations at a CV of 5%. Samples lacking an adequate number of respective events were excluded. All available markers were selected for clustering, with the exception of CD3/CD8/CD4 (for CD8^+^ T cells) and CD3/CD8/CD4/Foxp3 (for CD4^+^ T_Conv_ and CD4^+^ T_reg_). Perplexity was set to 70, theta to 0.5, and iterations set to 7500 for CD8^+^ T cells and CD4^+^ T_Conv_ and 2000 for CD4^+^ T_Reg._ Individual sample files from the viSNE analysis for each T cell population were downloaded from Cytobank as FCS files and concatenated into a single viSNE file using the R script (R-project.org) made available on the Cytobank platform and these files were then loaded into Cytobank for further analysis and visualization.

#### Clustering and predictive biomarker model development using CITRUS

To identify cell clusters predictive of water versus alcohol drinking animals, we utilized the CITRUS algorithm available within Cytobank. The same downsampled events used to generate each viSNE analysis for a respective T cell population were also used to perform the CITRUS modeling. Samples were assigned into water or alcohol-fed groups as appropriate and equal event sampling was selected for the analyses (i.e. 20,000 events/sample for CD8^+^ T cells and CD4^+^ T_Conv_ and 4,000 events/sample for CD4^+^ T_Reg_). The same channels used for each respective viSNE analyses were selected for clustering and cluster characterization was performed by abundance. Minimum cluster size was again set to 2% for CD8^+^ T cells and CD4^+^ T_Conv_, and 10% for T_Reg_. Cross validation folds were set to 10 and scales were selected for normalization. L1-Penalized Regression (LASSO) was selected as the association model for biomarker prediction (note: false discovery rate settings do not influence the analysis when using the LASSO model). This process was repeated 3 times for both viSNE maps of each respective T cell subset, yielding a total of 6 CITRUS runs each for CD8^+^ T cells, CD4^+^T_Conv_ and CD4^+^T_Reg_.

##### Selecting reproducible clusters of interest

For each T cell population, model error rate plots from the six CITRUS runs were examined to ensure an acceptable number of features and low cross validation error rate were generated. For each run the “CV.MIN” model was selected for analysis. Histograms from the “clusters” CITRUS output file were visually compared and clusters with similar phenotypes identified consistently across runs were selected for downstream analysis. If two or more of the selected clusters were identified as having “parent-child” relationships on the CITRUS feature plots, then only the parent cluster was used for downstream analysis unless the parent cluster split into more than one distinct branch, in which case only the child clusters were used for downstream analysis. The files for the selected clusters of interest for each T cell population were concatenated and their spatial locations visualized on their respective viSNE plots.

##### Phenotyping clusters of interest

Four multimodal data visualization strategies were employed to characterize clusters of interest that had high (hi) or low (lo) expression of a given marker. 1) observing the marker expression on the corresponding region of the cluster’s viSNE plot, 2) observing the marker expression on the corresponding region of the CITRUS marker plot, 3) observing histograms of marker expression produced by the CITRUS run, 4) observing Cytobank-generated heatmaps of concatenated median marker expression for each cluster (arcsinh transform) relative to the concatenated sample control. Clusters were assigned (hi) or (lo) expression of a given marker only if there was phenotypic agreement across 3 or more of these modalities.

##### viSNE visualization of validation cohort

Samples from the validation cohort for each T cell population were used to create new viSNE plots using identical settings as the original cohort, however only markers involved in characterizing the phenotype of the clusters of interest were selected for clustering the viSNE map. The results of the viSNE analysis for each T cell population were concatenated for the entire cohort as well as for the water and alcohol drinking experimental arms separately. Manual gating was performed on the viSNE plot within Cytobank to identify and isolate cell clusters with phenotypes analogous to the clusters of interest defined *via* CITRUS.

##### Analysis of validation cohort

Median marker expression heatmaps of each manually gated cluster on the validation cohort viSNE map were utilized to confirm that the phenotypes of these clusters were similar to those identified in the modeling cohort (arcsinh transformation using the concatenated total sample subset as a control). viSNE contour plots colored by density were utilized to visually compare cluster population density in the gated regions for water versus alcohol samples and confirm that the cluster behavior (increased or decreased) correlated with the behavior predicted by the CITRUS algorithm from the Modeling Cohort. Individual sample statistics (frequency) for each manually gated cluster were then exported from Cytobank and analyzed using Prism v9.0 software (GraphPad San Diego, CA) to quantitatively confirm changes to cluster frequency between alcohol and water drinking groups.

### Statistical analysis

For frequency analysis of exported manually gated clusters from the validation cohort, Prism v9.0 software (GraphPad San Diego, CA) was utilized. Outliers were identified and excluded using Grubb’s test with α=0.05. Data was then tested for Gaussian distribution using the Shapiro-Wilk normality test with α=0.05. Normally distributed data was compared using a two-tailed unpaired t test while non-normal data was compared with a two-tailed Mann-Whitney test. Data are expressed as mean ± SEM. The significance level was set to α=0.05.

## Results

### Absolute numbers of CD8^+^ T cells, CD4^+^ conventional T cells, and CD4^+^ Foxp3^+^ Treg are not different in water *vs*. alcohol-exposed mice

To determine the effects of chronic alcohol exposure on the magnitude and phenotype of CD4^+^ and CD8^+^ T cells, B6 mice were exposed to increasing concentrations of alcohol ad libitum in their drinking water over a period of 12 weeks as described in Materials and Methods (**Figure 1A**). Animals were subsequently euthanized and splenocytes were enumerated *via* flow cytometry using the gating strategy shown in **Figure 1B**. Results indicated no differences in the absolute numbers of bulk CD3^+^ T cells, bulk CD4^+^ T cells, bulk CD8^+^ T cells, Foxp3^-^ CD4^+^ conventional T cells (T_conv_) or Foxp3^+^ CD4^+^ regulatory T cells (T_reg_) (**Figure 1C**). These results indicate that prior to any immunologic or antigenic challenge, chronic exposure to alcohol does not change the magnitude of major T cells subsets in the mouse.

### Chronic alcohol exposure results in alterations in three CD4^+^ T_reg_ subpopulations

**Figure 2:**
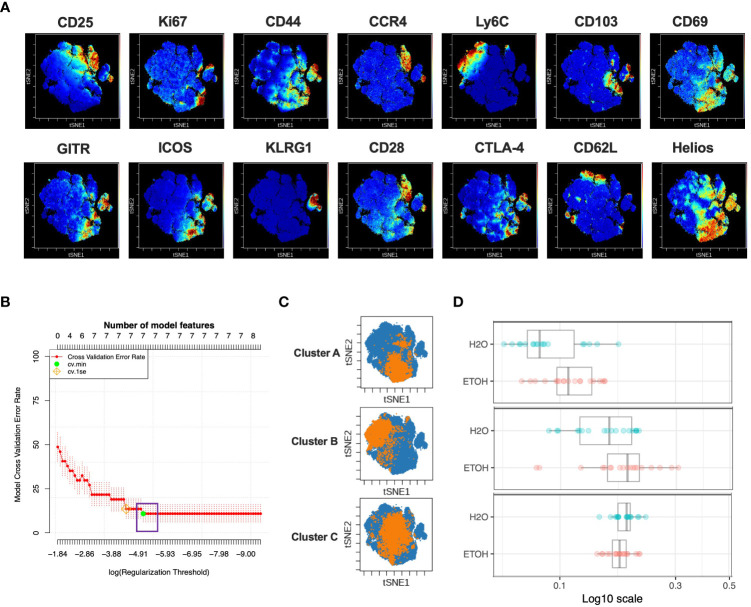
Combined CITRUS and viSNE analysis revealed three CD4^+^T_reg_ clusters associated with chronic alcohol exposure. **(A)** CD4^+^ T_reg_ subset samples from water- and alcohol-drinking mice were equally downsampled to 4000 events to detect 10% subpopulations with CV=5%. viSNE mapping was performed using all 14 available markers for clustering (CD3, CD4, CD8 and Foxp3 were excluded). Iterations were set to 2000, perplexity 70 and theta 0.5. **(B)** CITRUS analyses were performed on the events comprising each viSNE plot. Samples were grouped by alcohol versus water exposure, the same 14 channels used for viSNE were selected for clustering by abundance. LASSO modeling was selected, minimum cluster size was set to 10%, false discovery rate 1% (note: this is irrelevant for LASSO), cross validation folds 10, and scales were normalized. The CV.MIN output was selected from the LASSO analysis. A representative model error rate plot is show. **(C)** CITRUS replicates were compared using the “clusters-cv_min” output file histograms. Clusters that were altered in alcohol versus water drinkers were identified Clusters **(A–C)**. Representative clusters from one CITRUS run were concatenated and individually overlaid onto their corresponding viSNE plot shown in **(A)**. **(D)** Representative CITRUS feature plot corresponding to **(C)** showing differences in cluster frequency between alcohol and water exposed animals. E) The statistical frequencies of Clusters A (8.7 ± 0.6% *vs*. 6.8 ± 0.6%, p=0.04) and B (4.6 ± 0.4% *vs*. 2.8 ± 0.3%, p=0.002) were increased in alcohol exposed mice, while the frequency of Cluster C (2.4 ± 0.1% *vs*. 4.5 ± .2%, p <0.0001) was decreased. Data were tested for normality and compared with either t test or Mann-Whitney test.

Given the above results demonstrating that chronic alcohol exposure did not impact the quantity of T cell subsets, we next asked whether chronic alcohol exposure impacted the quality of T cell subsets. To identify changes in the CD4^+^T_reg_ compartment during chronic exposure to alcohol, CD3^+^ CD4^+^ CD8^-^ Foxp3^+^ T_reg_ were gated using FlowJo. All samples, both alcohol- and water-drinking, were downsampled and clustered by all 14 available markers to generate a viSNE map visualizing subpopulations as small as 10% of CD4^+^T_reg_ (**Figure 2A**). This process was repeated once to generate a second viSNE map from randomly sampled events.

In order to identify CD4^+^T_reg_ subpopulations as small as 10% that were associated with chronic exposure to alcohol, the LASSO association model of the CITRUS algorithm was performed with the same downsampled events and clustering channels used to create the corresponding viSNE map to allow the results of CITRUS to be visualized with viSNE. Three CITRUS models were generated in this manner for both CD4^+^T_reg_ viSNE plots, yielding six total models generated for this subset, all of which demonstrated a sufficient number of features and acceptably low error rate (**Figure 2B**). Given that CITRUS models can differ between replicates when more than one subpopulation is by itself adequately predictive of an endpoint, we only selected phenotypically-identical clusters that were present in all six replicates for downstream use in order to guard against false positives and restrict our analyses to a manageable number of subpopulations. This strategy identified three CITRUS clusters with phenotypes consistently present across all iterations, and these were then concatenated and overlaid onto their respective regions of the viSNE map (**Figure 2C**). Clusters A and B were consistently increased in alcohol exposed animals, while Cluster C was decreased (**Figure 2D**).

**Figure 3:**
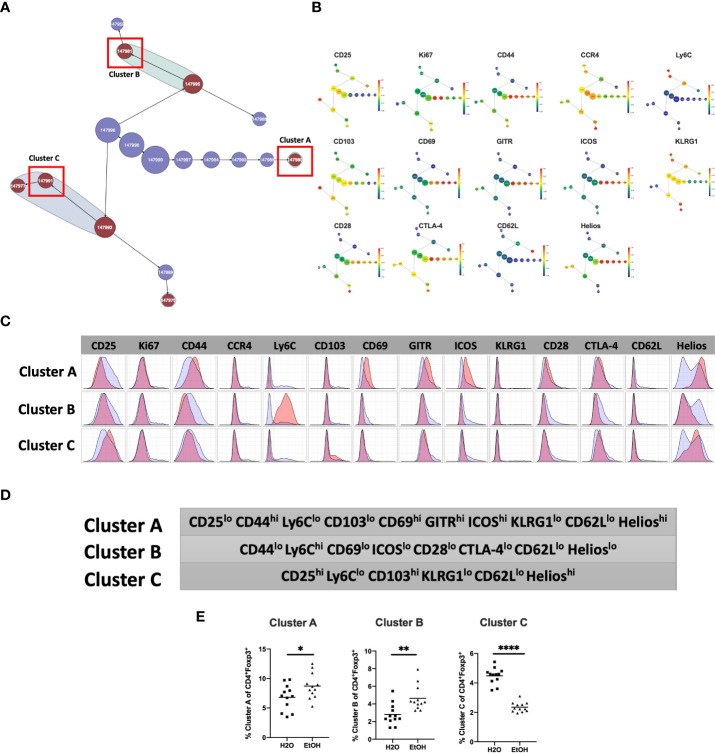
Phenotypic analysis of three CD4^+^ T_reg_ clusters that are significantly different in water- *vs*. alcohol-exposed mice. Following CITRUS modeling and identification of three CD4^+^T_Reg_ clusters predictive of alcohol drinking mice, phenotypic cluster characterization was performed by assessing **(A)**, marker expression on the viSNE map with the overlaid clusters, **(B)**, CITRUS clusters of interest shown in the feature plots in **(A)** were compared to expression on corresponding the heatmap marker plots in **(B, C)** CITRUS marker histogram expression was visualized where blue represents background expression and red represents expression within the cluster of interest. The final phenotypic characterization profile assigned to the three clusters of interest is shown in **(D, E)**. Manual gating demonstrated that the statistical frequencies of Clusters **(A)** (8.7 ± 0.6% *vs*. 6.8 ± 0.6%, p=0.04) and **(B)** (4.6 ± 0.4% *vs*.2.8 ± .3%, p=0.002) were increased in alcohol exposed mice, while the frequency of Cluster **(C)** (2.4 ± 0.1% *vs*. 4.5 ± 0.2%, p <0.0001) was decreased. Data were tested for normality and compared with either t test or Mann-Whitney test. *p<0.05, **p<0.01, ****p<0.0001.

We then employed a multimodal visualization strategy in order to phenotype these clusters First, we assessed marker expression on the corresponding region (**Figure 2C**) of a cluster’s viSNE plot (**Figure 3A**). In addition, marker expression on the corresponding region (**Figure 3A**) of the CITRUS marker plots (**Figure 3B**) and histograms of marker expression produced by the CITRUS run (**Figure 3C**) were assessed. Cluster A was determined to be CD25^lo^CD44^hi^Ly6C^lo^CD103^lo^CD69^hi^GITR^hi^ICOS^hi^KLRG1^lo^CD62L^lo^Helios^hi^, Cluster B was determined to be CD44^lo^Ly6C^hi^CD69^lo^ICOS^lo^CD28^lo^CTLA-4^lo^CD62L^lo^Helios^lo^, and Cluster C was determined to be CD25^hi^Ly6C^lo^CD103^hi^KLRG1^lo^CD62L^lo^Helios^hi^ (**Figure 3D**).

To confirm the generalizability of our findings, we tested for the presence of similar differences between in Treg subpopulations between alcohol and water exposed mice in a separate validation cohort. Manual gating was then performed within Cytokbank on the validation cohort viSNE map to locate regions with corresponding phenotypes to CD4^+^T_reg_ Clusters A, B and C (**Figures 3A**, **C**) (gating strategy shown in **Supplemental Figure 1**). Frequencies of these manually gated regions were exported for each sample and analyzed using traditional statistical techniques and quantitatively verified that Clusters A and B were significantly increased, and C significantly decreased, in alcohol-fed relative to water-fed animals as predicted by the CITRUS model (**Figure 3E**).

### Chronic alcohol exposure results in decreased frequency of Ki67^lo^CD44^lo^CCR4^lo^Ly6C^lo^CD69^lo^KLRG1^lo^CD28^lo^CD62L^hi^Helios^hi^ CD4^+^T_Conv_

**Figure 4:**
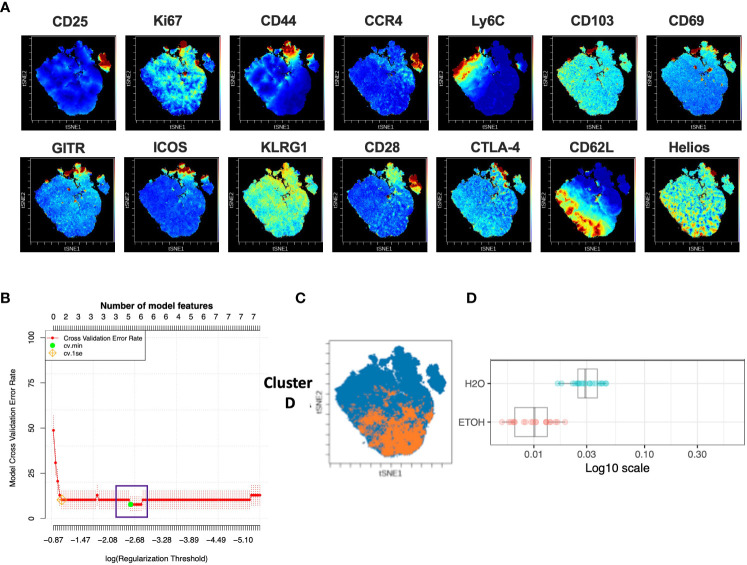
Combined CITRUS and viSNE analysis revealed one CD4^+^T_Conv_ cluster associated with alcohol-drinking mice. **(A)** CD4^+^T_Conv_ subset samples from the modeling cohort were equally downsampled to 20000 events to detect 2% subpopulations with CV=5%. 1 water drinking sample was excluded due to insufficient event count. viSNE mapping was performed using all 14 available markers for clustering (CD3, CD4, CD8 and Foxp3 were excluded). Iterations were set to 7500, perplexity 70 and theta 0.5. This process was repeated to create two total viSNE maps, one of which was concatenated and displayed above. **(B)** CITRUS analyses was performed on the events comprising each viSNE plot. Samples were grouped by alcohol versus water exposure, the same 14 channels used for viSNE were selected for clustering by abundance. LASSO modeling was selected, minimum cluster size was set to 2%, false discovery rate 1% (note: this is irrelevant for LASSO), cross validation folds 10, and scales were normalized. The CV.MIN output was selected from the LASSO analysis. This process was repeated 3 times for each of the two viSNE maps yielding 6 total CITRUS replicates. A representative model error rate plot is show. **(C)** 6 CITRUS replicates were compared using the “clusters-cv_min” output file histograms. 1 cluster predictive of alcohol versus water drinkers was present across all replicates Cluster **(D)** and was selected for downstream analyses. Representative clusters from one CITRUS run were concatenated and individually overlaid onto their corresponding viSNE plot shown in **(A)**. **(D)** Representative CITRUS feature plot corresponding to **(C)** showing differences in cluster frequency between alcohol and water exposed animals.

Analysis of the CD4^+^T_conv_ compartment to identify subpopulations predictive of chronic alcohol exposure was performed in a manner analogous to that of CD4^+^T_reg_ above. The CD4^+^T_conv_ subset of the modeling cohort underwent viSNE analysis and mapping to detect subpopulations as small as 2% (**Figure 4A**). CITRUS analysis was performed and again generated models with sufficient numbers of features and acceptably low error rate (**Figure 4B**). We identified one CITRUS cluster- Cluster D - (**Figure 4C**) with a phenotype consistently present across all CITRUS iterations and whose frequency was decreased in alcohol exposed animals (**Figure 4D**).

**Figure 5:**
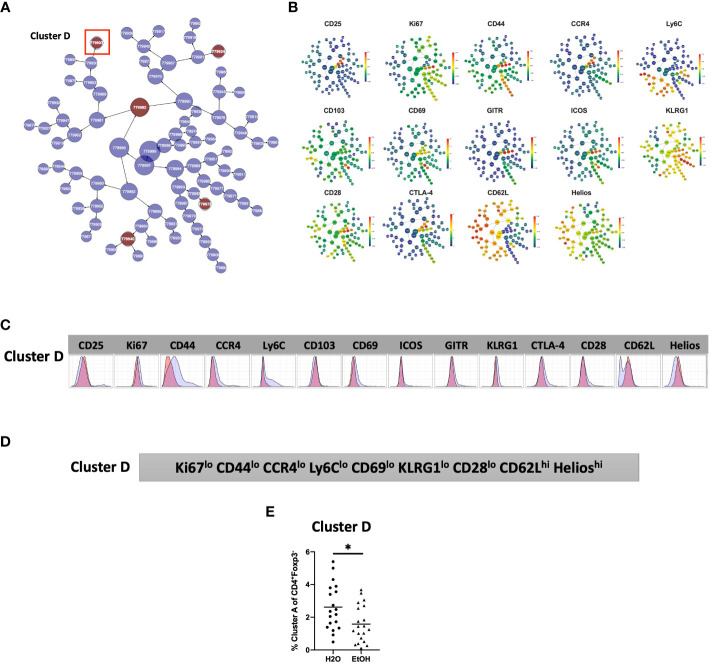
Phenotypic analysis of one CD4^+^ T_Conv_ cluster that is significantly different in alcohol-exposed mice. Following CITRUS modeling and identification of three CD4^+^T_Conv_ clusters predictive of alcohol drinking mice, phenotypic cluster characterization was performed by assessing **(A)** marker expression on the viSNE map with the overlaid clusters, **(B)** CITRUS clusters of interest shown in the feature plots in **(A)** were compared to expression on corresponding the heatmap marker plots in **(B, C)** CITRUS marker histogram expression was visualized where blue represents background expression and red represents expression within the cluster of interest. The final phenotypic characterization profile assigned to the cluster of interest is shown in **(D)**. **(E)** Manual gating demonstrated that the frequency of cells within Cluster **(D)** (1.6 ± 0.3% *vs*. 2.6 ± 0.3%, p=0.01) was statistically significantly decreased in alcohol exposed mice relative to water exposed. Data were tested for normality and compared using t test. *p<0.05.

The phenotype of cells in CD4^+^T_Conv_ Cluster D was then interrogated by assessing marker expression on the corresponding region (**Figure 4C**) of the cluster’s viSNE plot (**Figure 5A**), marker expression on the corresponding region (**Figure 5A**) of the CITRUS marker plots (**Figure 5B**), and histograms of marker expression produced by the CITRUS run (**Figure 5C**). Based on these, Cluster D within the CD4^+^ T cell compartment was determined to be Ki67^lo^CD44^lo^CCR4^lo^Ly6C^lo^CD69^lo^KLRG1^lo^CD28^lo^CD62L^hi^Helios^hi^ (**Figure 5D**).

To confirm the generalizability of our findings, we again performed manual gating within Cytobank on a validation cohort to identify a region on the viSNE map with a similar phenotype to CD4^+^T_Conv_ Cluster D (**Supplemental Figure 2**). Statistical analysis demonstrated that the frequency of cells within CD4^+^T_Conv_ Cluster D was significantly decreased in alcohol exposed mice (**Figure 5E**).

### Chronic alcohol exposure results in decreased frequency of CD25^lo^ CD44^lo^ Ly6C^lo^ CD103^hi^ CD8^+^ T cells

**Figure 6:**
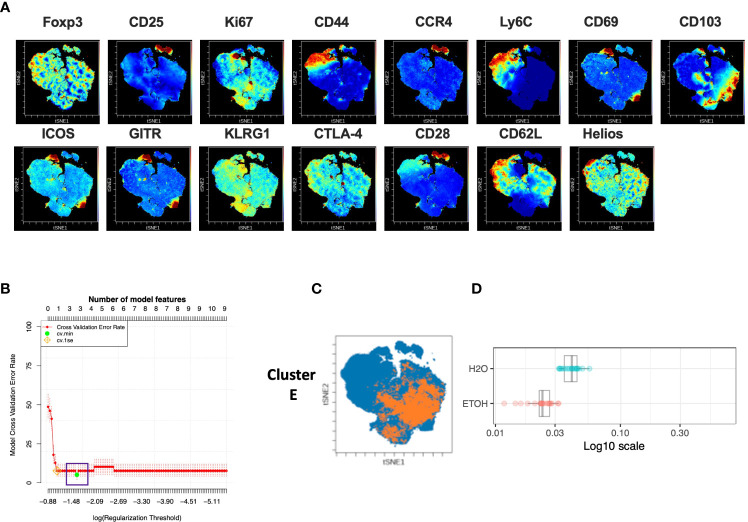
Combined CITRUS and viSNE analysis revealed one CD8^+^ T cell cluster associated with alcohol-drinking mice. **(A)** CD8^+^T_cell_ subset samples from the modeling cohort were equally downsampled to 20000 events to detect 2% subpopulations with CV=5%. 1 water drinking sample was excluded due to insufficient event count. viSNE mapping was performed using all 15 available markers for clustering (CD3, CD4 and CD8 were excluded). Iterations were set to 7500, perplexity 70 and theta 0.5. This process was repeated to create two total viSNE maps, one of which was concatenated and displayed above. **(B)** CITRUS analyses was performed on the events comprising each viSNE plot. Samples were grouped by alcohol versus water exposure, the same 14 channels used for viSNE were selected for clustering by abundance. LASSO modeling was selected, minimum cluster size was set to 2%, false discovery rate 1% (note: this is irrelevant for LASSO), cross validation folds 10, and scales were normalized. The CV.MIN output was selected from the LASSO analysis. This process was repeated 3 times for each of the two viSNE maps yielding 6 total CITRUS replicates. A representative model error rate plot is show. **(C)** 6 CITRUS replicates were compared using the “clusters-cv_min” output file histograms. 1 cluster predictive of alcohol versus water drinkers was present across all replicates Cluster (E) and was selected for for downstream analyses. Representative clusters from one CITRUS run were concatenated and individually overlaid onto their corresponding viSNE plot shown in **(A)**. **(D)** Representative CITRUS feature plot corresponding to **(C)** showing differences in cluster frequency between alcohol and water exposed animals.

Analysis of the CD8^+^ T cell compartment to identify subpopulations predictive of chronic alcohol exposure was performed in a manner analogous to that of CD4^+^T_reg_ and CD4^+^T_conv_ above. The CD8^+^ T cell subset of the modeling cohort underwent viSNE analysis and mapping to detect subpopulations as small as 2% (**Figure 6A**). CITRUS analysis was performed and again generated models with sufficient numbers of features and acceptably low error rate (**Figure 6B**). We identified one CITRUS cluster- Cluster E - (**Figure 6C**) with a phenotype consistently present across all CITRUS iterations and the frequency of which was decreased in alcohol exposed animals (**Figure 6D**).

**Figure 7:**
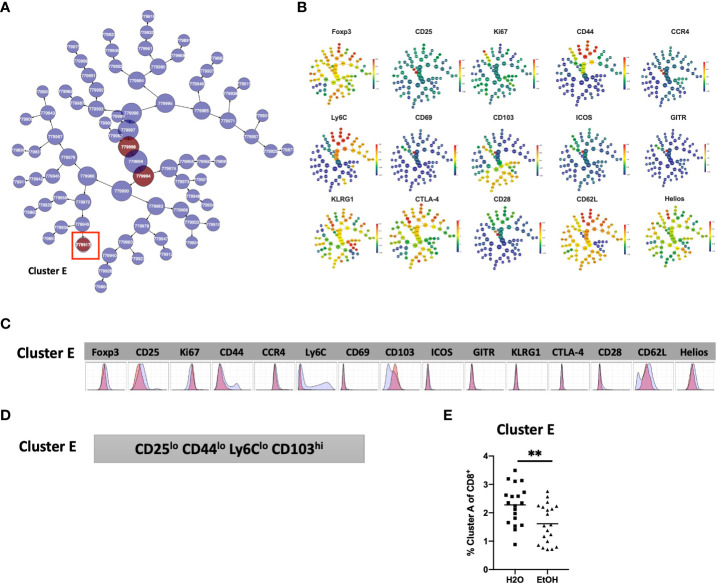
Phenotypic analysis of one CD8^+^ T cell cluster that is significantly different in alcohol exposed mice. Following CITRUS modeling and identification of one CD8^+^ T cell clusters predictive of alcohol drinking mice, phenotypic cluster characterization was performed **(A)**, assessing marker expression on the viSNE map with the overlaid clusters, **(B)** CITRUS clusters of interest shown in the feature plots in **(A)** were compared to expression on corresponding the heatmap marker plots in **(B, C)** CITRUS marker histogram expression was visualized where blue represents background expression and red represents expression within the cluster of interest. The final phenotypic characterization profile assigned to the cluster of interest is shown in **(D)**. **(E)** The frequency of Cluster E (1.6 ± 0.2% *vs*. 2.3 ± 0.2%, p=0.005) was significantly decreased in alcohol exposed mice relative to water exposed. Data were tested for normality and compared a *t* test.

The phenotype of cells in Cluster E was then interrogated by assessing marker expression on the corresponding region (**Figure 6C**) of the cluster’s viSNE plot (**Figure 7A**), assessing marker expression on the corresponding region of the CITRUS marker plots (**Figure 7B**), and histograms of marker expression produced by the CITRUS run (**Figure 7C**). Cluster E within the CD8^+^ T cell compartment was determined to be CD25^lo^CD44^lo^Ly6C^lo^CD103^hi^ (**Figure 7D**).

To confirm the generalizability of our findings, manual gating was again performed within Cytobank to identify a region on the viSNE map with a similar phenotype to CD8^+^ T cell Cluster E (gating strategy shown in **Supplemental Figure 3**). Statistical analysis verified that the frequency of Cluster E within the CD8^+^ T cell compartment was significantly decreased in alcohol exposed mice, also as predicted by the CITRUS model (**Figure 7E**).

## Discussion

High dimensional, single-cell flow cytometry analysis techniques continue to evolve and provide increased ability to characterize rare immune cell populations. Here, we have presented an application of algorithms available through the Cytobank platform to perform an exploratory characterization of rare T cell populations that are present in mice which are chronically exposed to alcohol. We used an iterative series of viSNE and CITRUS analyses to identify subpopulations within the CD8^+^, CD4^+^T_Conv_ and CD4^+^T_reg_ compartments, the frequencies of which are reproducibly different between alcohol- and non-alcohol-exposed animals. This was followed by a multimodal approach to phenotype these clusters and then, finally, validation of these findings in a testing cohort by identifying analogous subpopulations with identical frequency differences between alcohol and water-fed mice. Specifically, we identified **CD25^lo^CD44^lo^Ly6C^lo^CD103^hi^** CD8^+^ T cell, **Ki67^lo^CD44^lo^CCR4^lo^Ly6C^lo^CD69^lo^KLRG1^lo^CD28^lo^CD62L^hi^Helios^hi^** CD4^+^T_conv_, and **CD25^hi^Ly6C^lo^CD103^hi^KLRG1^lo^CD62L^lo^Helios^hi^** CD4^+^T_reg_ populations that were decreased in alcohol exposed animals. Conversely, two CD4^+^T_reg_ populations – **CD25^lo^CD44^hi^Ly6C^lo^CD103^lo^CD69^hi^GITR^hi^ICOS^hi^KLRG1^lo^CD62L^lo^Helios^hi^**, and **CD44^lo^Ly6C^hi^CD69^lo^ICOS^lo^CD28^lo^CTLA-4^lo^CD62L^lo^Helios^lo^** were both increased in alcohol-exposed relative to water-fed animals.

Within the CD8^+^ T cell compartment, the identification of a population with low expression of both CD44 and Ly6C is strongly suggestive of a naïve phenotype, as both these markers are known to be upregulated in antigen-experienced T cells (42–44), with Ly6C specifically associated with CD8^+^ central memory (CD8^+^T_CM_) cells (45). Similarly, CD25, the receptor for IL-2, is recognized as a general marker of activation and antigen-experience (46) and low expression of CD25 in this setting is likely further indicative of a naïve phenotype. CD103 is a well-described marker of tissue homing that allows cells to bind E-cadherin expressed on peripheral epithelial tissues (47). In this context, expression of CD103 on CD8^+^ T cells is a characteristic feature of the tissue resident memory phenotype (CD8^+^T_RM_) and distinguishes these cells from effector memory CD8^+^ T cells (CD8^+^T_EM_), both of which lack expression of CD62L (L-selectin) to facilitate retention in secondary lymphoid organs. Similar to the CD8^+^ compartment, our analysis of the CD4^+^T_Conv_ compartment also revealed loss of a subpopulation that shared low expression of CD44 and Ly6C in addition to low expression of proliferative marker Ki67 (48), co-stimulatory molecule CD28 (49), activation marker CD69 (50) and terminal differentiation marker KLRG1 (51). CCR4, a chemokine receptor associated with Th2 polarization and cutaneous T cell migration (52) as well as cell retention in inflamed tissues (53) was also decreased in this subpopulation. Conversely, this CD4^+^T_Conv_ cluster showed high expression of CD62L, rather than CD103, in addition to high expression of Helios, which has been shown to be critical for the activation of naïve T cells (54, 55). Together, these findings suggest that chronic alcohol exposure in mice leads to loss of naïve subsets of CD8^+^ T cells and CD4^+^T_Conv_ cells that specifically possess CD103^+^ and Helios^+^ phenotypes, respectively. Given that loss of naïve T cell subsets is a known consequence of chronic alcohol exposure (6, 14), the data presented here both confirm this conclusion as well as provide more detailed phenotypic characterization of this population that may inform future mechanistic studies into the sequalae of chronic alcohol exposure in the adaptive immune system.

In the CD4^+^T_reg_ compartment, chronic alcohol exposure led to altered frequencies of Clusters A, B and C, all of which were found to demonstrate phenotypic characteristics that are largely consistent with those known to describe eT_reg_, a highly proliferative T_Reg_ subset that migrates to, and suppresses, end-organ inflammation (56). eT_reg_ are defined as CD62L^-^CD44^+^ (57) and express increased ICOS (56) and decreased CD25 (58) given their dependence on TCR stimulation (59), rather than IL-2 (58), for maintenance. eT_reg_ generally show increased markers of T_reg_ activation such as GITR (60), Helios (61), CTLA-4 (56), and KLRG1 (62), as they are unidirectionally derived from the activation and differentiation of the quiescent cT_reg_ subset (62). cT_reg_ conversely possess a CD62L^+^CD44^-^ phenotype (57), depend on IL-2 signaling *via* CD25 (58) and suppress inflammation in secondary lymphoid organs in addition to serving as an eT_reg_ precursor pool (62). The transition from cT_reg_ to eT_reg_ characteristically involves loss of Ly6C, indicative of senescence (62), and gain of CD69 and/or CD103. As described by Toomer 5 CD69^-^ CD103^-^ eT_Reg_ likely represent a transitional phenotype between cT_reg_ and eT_reg_, while CD69^+^ and CD103^+^ eT_reg_ are two distinct activated subsets (62). Collectively, these data suggest that our findings in the CD4^+^T_reg_ compartment of alcohol exposed mice may represent increased frequency of a CD69^+^ eT_reg_ population (Cluster A), along with decreased frequency of a CD103^+^ eT_reg_ population also expressing increased CD25 (Cluster C). Interestingly, CD4^+^T_reg_ Cluster B demonstrated features suggestive of both cT_reg_ (i.e. CD44^lo^Ly6C^hi^) and eT_reg_ (i.e. CD62L^lo^) and therefore, given its increased frequency in alcohol exposed animals, may be a manifestation of a transitional phenotype between the two.

The increased computational power offered to investigators by automated cytometry analyses is still not without notable limitations. Both viSNE and CITRUS effectively require investigators to decide what minimum subpopulation frequency they wish to target and with what statistical power. We considered the selection of 10% subpopulations for CD4^+^T_Reg_ and 2% subpopulations for CD4^+^T_Conv_ and CD8^+^ T cells reasonable given our interest in rare events (41), but any cut-off will inherently bias the results simply by the nature of the algorithms the software employs. However, our use of a validation cohort to test the predictions of the modeling cohort strengthens the generalizability of our findings and guards against the risk of over-fitting of CITRUS models to the underlying data. In addition, we employed a conservative approach as to which CITRUS clusters were selected for downstream analysis, increasingly the likelihood that our findings are generalizable to alcohol exposed mice, but at the cost of decreased sensitivity to identify all possible subpopulations differing in alcohol exposed animals. The current study is also limited by the fact that only splenocytes were analyzed. Because tissue microenvironment is known to affect T cell phenotype, it is possible that the T cell phenotypes we identified to be differentially expressed in the spleens of water- *vs*.- alcohol drinking animals may not be observed in other tissues. In prior analyses we have found that phenotypes in the spleen closely mirror those in the peripheral blood, given the large amount of blood circulating through the spleen. Follow up studies are planned to analyze other tissue microenvironments, such as the peritoneal lymph nodes, peritoneal exudate cells, the bone marrow, and intestinal lymphocytes.

In sum, using a series of machine-driven, multiparameter flow cytometry analyses strategies, we have successfully identified rare populations of naïve Helios^+^ CD4^+^T_conv_ and naïve CD103^+^ CD8^+^ splenic T cells that are decreased in chronically alcohol exposed mice versus water-fed controls, as well as increased CD69^+^ and decreased CD103^+^ eT_reg_ subsets in conjunction with increased frequency of a population that may represent a transitional phenotype between cT_reg_ and eT_reg_. These data provide further resolution into the character of decreased naïve T cell populations known to be present in alcohol exposed mice, as well as describe alterations in effector regulatory T cell phenotypes as consequence of chronic exposure to alcohol that are worthy of future study.

## Data availability statement

The raw data supporting the conclusions of this article will be made available by the authors, without undue reservation.

## Ethics statement

This study was approved by the Emory University Institutional Animal Care and Use Committee (IACUC) [Protocol: PROTO201800161].

## Author contributions

Conceptualization: MF and CC. Data curation: CP and MG. Formal analysis: CP and MG. Funding acquisition: MF and CC. Investigation: CP and MG. Project administration: CC and MF. Supervision: CC and MF. Visualization: CP, MG, and MF. Writing – original draft: CP and MF. Writing – reviewing and editing: MF and CC. All authors contributed to the article and approved the submitted version.

## Author disclaimer

The views expressed in this article reflect the results of research conducted by the author and do not necessarily reflect the official policy or position of the Department of the Navy, Department of Defense, nor the United States Government.” LT CP is a military service member or federal/contracted employee of the United States government. This work was prepared as part of his official duties. Title 17 U.S.C. 105 provides that `copyright protection under this title is not available for any work of the United States Government.’ Title 17 U.S.C. 101 defines a U.S. Government work as work prepared by a military service member or employee of the U.S. Government as part of that person’s official duties.
