# Supplementary material for: Impact of chronic alcohol exposure on conventional and regulatory murine T cell subsets
Source: Front Immunol. 2023 Mar 17;14:1142614. doi: 10.3389/fimmu.2023.1142614 (PMC10063870; doi:10.3389/fimmu.2023.1142614)
Supplement: Supplementary file 1 [file DataSheet_1.pdf]

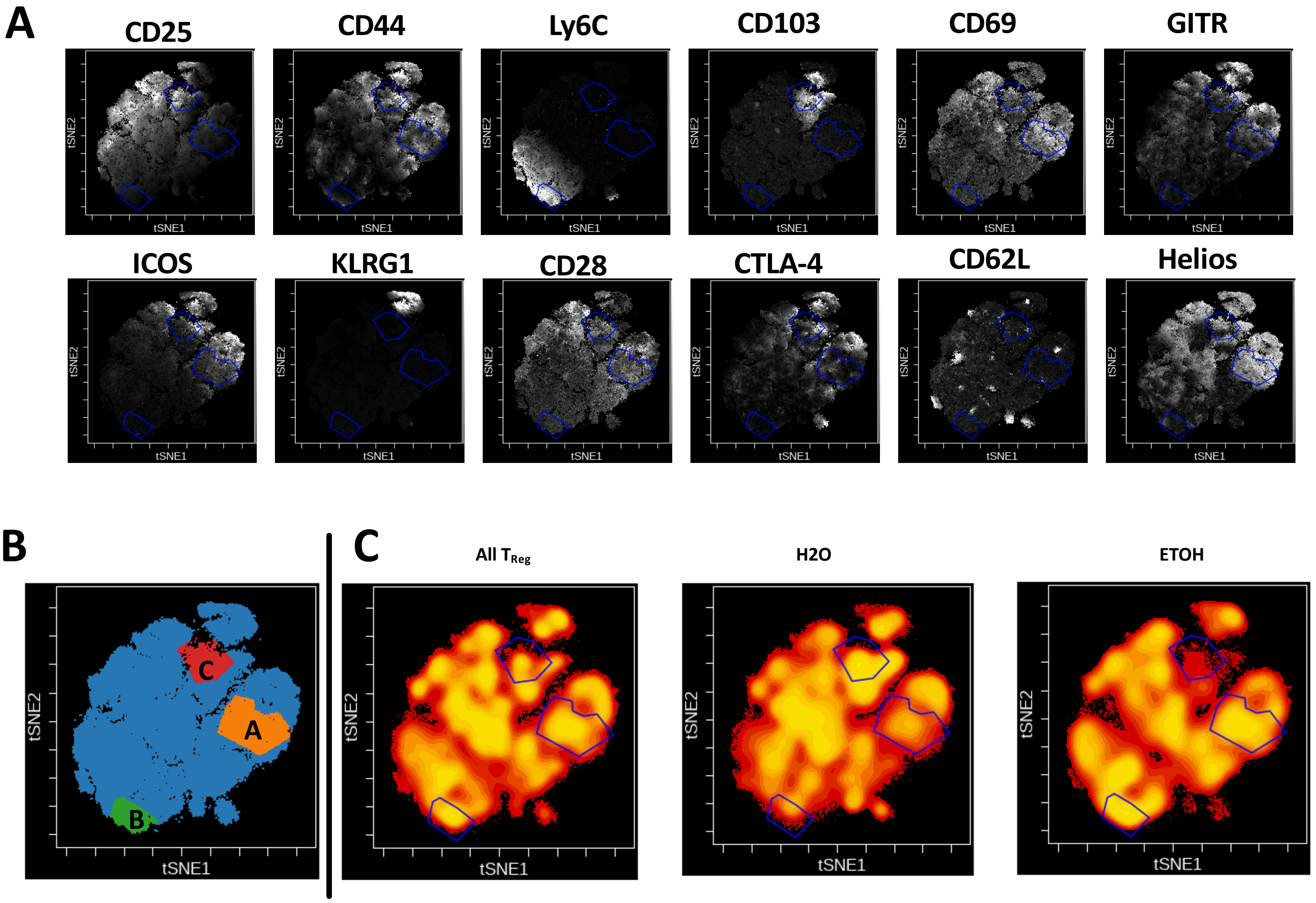

Supplemental Figure 1. Gating strategy for analysis of a validation cohort for changes in Foxp3^{+} T_{Reg} in alcohol drinking mice predicted by CITRUS modeling. The validation cohort (n=20/group) was mapped with viSNE using the 12 markers determined to characterize Clusters A, B and C in the modeling cohort for clustering. Downsampling was set to 4000 events/sample, 2000 iterations, 70 perplexity, theta 0.5. Samples with less than 4000 T_{Reg} events were excluded with 24 (n=12 water and n=12 alcohol) included in the final analysis. **A)** Events from the viSNE map are concatenated and displayed for the 12 clustering markers. Manual gating was performed within Cytobank on the viSNE map to isolate populations with phenotypes mirroring that of the 3 clusters identified in the modeling cohort. **B)** The gated regions for Clusters A, B and C as identified in (A) are shown as overlaid on the viSNE plot. **C)** Concatenated contour plots colored by density showing the gated areas correspond to discrete high-density regions within the viSNE plot.

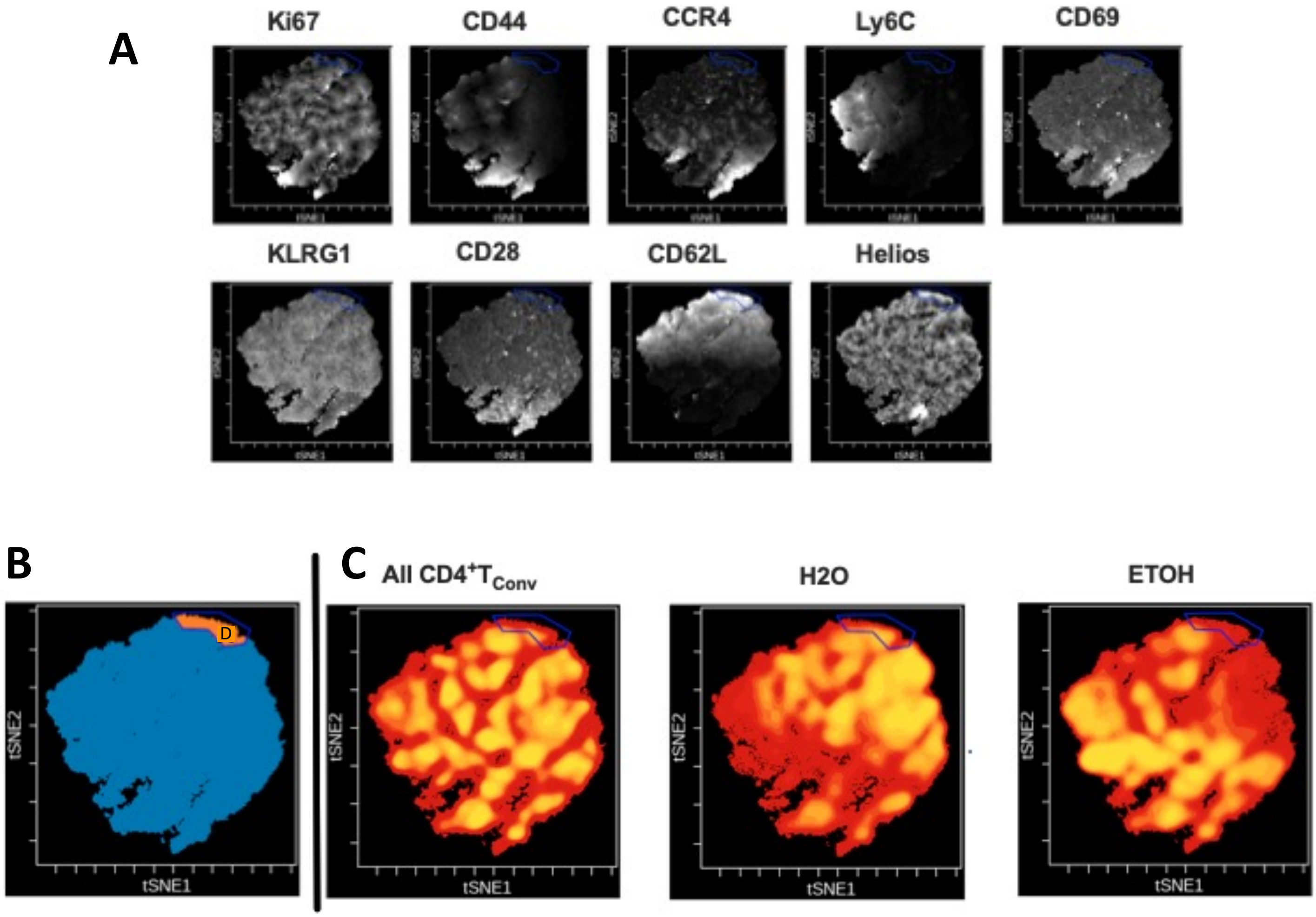

Supplemental Figure 2. Gating strategy for analysis of a validation cohort for changes in CD4^{+}T$_{conv}$ in alcohol drinking mice predicted by CITRUS modeling. The validation cohort (n=20/group) was mapped with viSNE using the 9 markers determined to characterize Cluster D for clustering. Downsampling was set to 20000 events/sample, 7500 iterations, 70 perplexity, theta 0.5. Samples with less than 20000 CD4^{+}T$_{Conv}$ events were excluded with 39 (n=19 water and n=20 alcohol) included in the final analysis. **A)** Events from the viSNE map are concatenated and displayed for the 9 clustering markers. Manual gating was performed within Cytobank on the viSNE map to isolate a population with a phenotype mirroring that of the cluster identified in the modeling cohort. **B)** The gated region for Clusters D in (A) are shown as overlaid on the viSNE plot. **C)** Concatenated contour plots colored by density showing the gated area corresponded to a discrete high-density region within the viSNE plot.

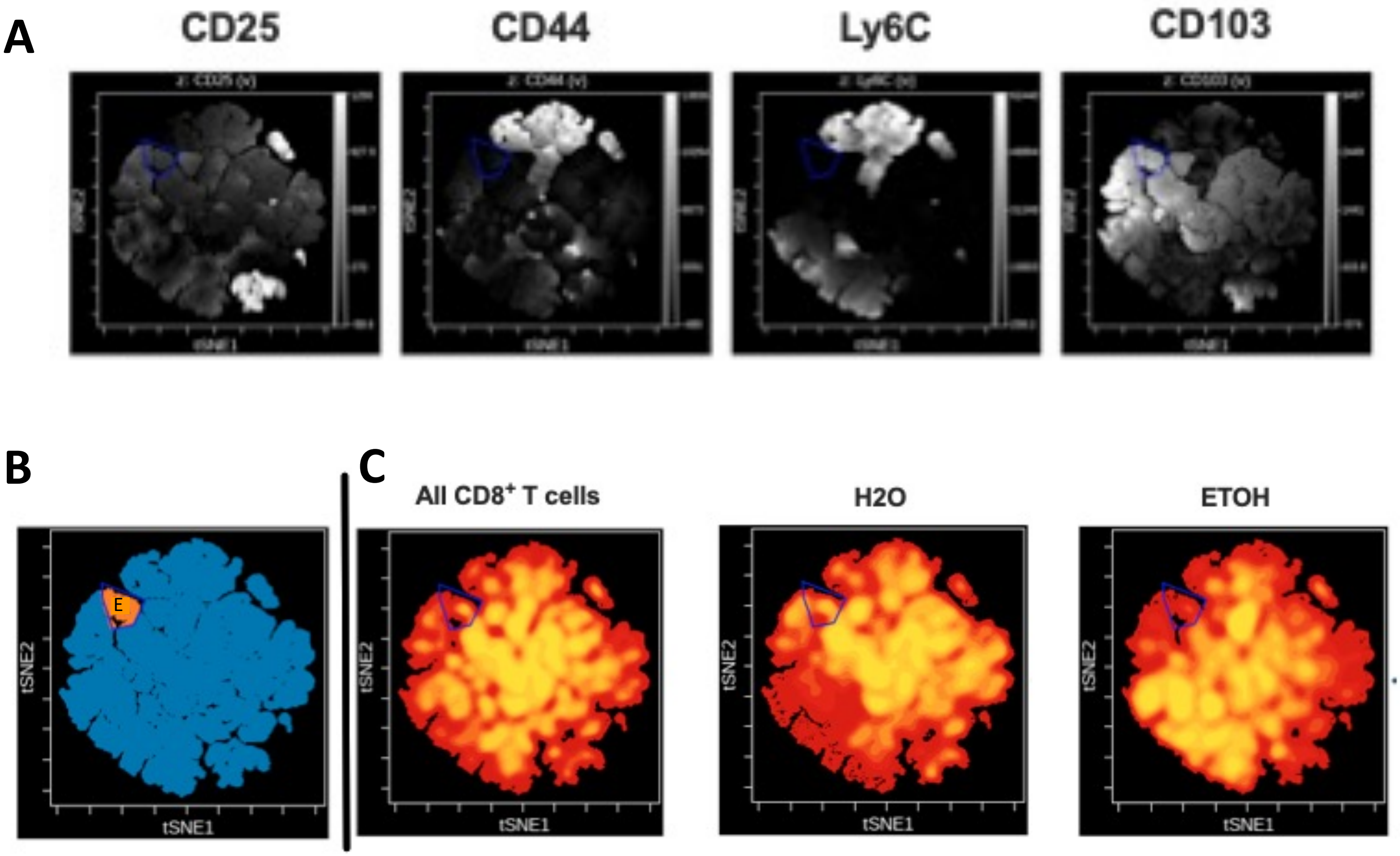

Supplemental Figure 3. Gating strategy for analysis of a validation cohort for changes in CD8$^+$ T cells in alcohol drinking mice predicted by CITRUS modeling. The validation cohort (n=20/group) was mapped with viSNE using the 4 markers determined to characterize Cluster E for clustering. Downsampling was set to 20000 events/sample, 7500 iterations, 70 perplexity, theta 0.5. Samples with less than 20000 CD8$^+$ T cell events were excluded with 39 (n=19 water and n=20 alcohol) included in the final analysis. **A)** Events from the viSNE map are concatenated and displayed for the 4 clustering markers. Manual gating was performed within Cytobank on the viSNE map to isolate a population with a phenotype mirroring that of the cluster identified in the modeling cohort. **B)** The gated region for Clusters E in (A) are shown as overlaid on the viSNE plot. **C)** Concatenated contour plots colored by density showing the gated area corresponded to a discrete high-density region within the viSNE plot.
